# Scalable and Accurate ECG Simulation for Reaction-Diffusion Models of the Human Heart

**DOI:** 10.3389/fphys.2018.00370

**Published:** 2018-04-20

**Authors:** Mark Potse

**Affiliations:** ^1^CARMEN Research Team, Inria Bordeaux Sud-Ouest, Talence, France; ^2^Institut de Mathématiques de Bordeaux, UMR 5251, Université de Bordeaux, Talence, France; ^3^IHU Liryc, Electrophysiology and Heart Modeling Institute, Foundation Bordeaux Université, Pessac-Bordeaux, France

**Keywords:** numerical modeling, electrocardiogram, high-performance computing, reaction-diffusion model, bidomain model, lead fields

## Abstract

Realistic electrocardiogram (ECG) simulation with numerical models is important for research linking cellular and molecular physiology to clinically observable signals, and crucial for patient tailoring of numerical heart models. However, ECG simulation with a realistic torso model is computationally much harder than simulation of cardiac activity itself, so that many studies with sophisticated heart models have resorted to crude approximations of the ECG. This paper shows how the classical concept of electrocardiographic lead fields can be used for an ECG simulation method that matches the realism of modern heart models. The accuracy and resource requirements were compared to those of a full-torso solution for the potential and scaling was tested up to 14,336 cores with a heart model consisting of 11 million nodes. Reference ECGs were computed on a 3.3 billion-node heart-torso mesh at 0.2 mm resolution. The results show that the lead-field method is more efficient than a full-torso solution when the number of simulated samples is larger than the number of computed ECG leads. While the initial computation of the lead fields remains a hard and poorly scalable problem, the ECG computation itself scales almost perfectly and, even for several hundreds of ECG leads, takes much less time than the underlying simulation of cardiac activity.

## 1. Introduction

The electrocardiogram (ECG) is one of the most common tools in present-day medicine, yet its relation with the molecular biology of the heart is still poorly understood. The ECG witnesses the collective activity of about a million current-generating transmembrane proteins in each of the heart's muscle cells (Hille, 2001). Many of these proteins have been identified and their actions have been captured in mathematical models that predict their collective behavior on the scale of a cell (Noble and Rudy, 2001). By coupling millions of these membrane models one can create a model of whole-heart electrophysiology. Such models generate crucial insights in the functional effects of molecular-level changes, allowing for example to predict dangerous side effects of new drug designs (Passini et al., 2017) or to understand how cardiac ion-channel mutations influence cardiac rhythm disorders (Gima and Rudy, 2002). Moreover, from their results one can compute the corresponding ECG and predict how lab results on subcellular components would translate to everyday practice (Hoogendijk et al., 2010; Keller et al., 2012; Zemzemi et al., 2013).

Such realistic models are large and, when run on a single processor, would take days to simulate just one heartbeat. Fortunately the problem can be expressed in such a way that the work may be spread over many processors with little communication between them. Therefore, these computations are said to scale very well, meaning that they run almost twice as fast every time the number of processors is doubled (Vázquez et al., 2011). This makes them suitable for use on large-scale parallel computers, allowing models to run in nearly real time (Niederer et al., 2011b; Richards et al., 2013).

Simulation of a realistic ECG from the results of such a numerical heart model is much harder, because the electrical current generated by the heart meets a different conductivity at each point in the torso. As a result, each point influences the potential everywhere else, so to find the potential anywhere one must solve it everywhere at the same time.

Numerically this means that a large system of linear equations must be solved, one for each point in the torso model. These problems are harder when they are larger and require frequent communication between the processors in a parallel computer. This means that they cannot be solved much faster by using more processors. Therefore, ECG computation is becoming a bottleneck, limiting both the speed and the spatial resolution of our models.

To avoid this problem many researchers have used simplified torso models, resulting in a less accurate ECG. A solution that can avoid such a sacrifice is to simulate the ECG using an electrocardiographic concept named a lead field. This allows the problem to be split into a hard (poorly scaling) part and an easy (well scaling) part. The hard part is solved only once for each ECG lead, while the easy part is run repeatedly for each time step in a simulation and for multiple simulations on the same geometry. This approach has been used by several authors, but generally with simplified heart models (Pezzuto et al., 2017) or, again, with simplified torso models (Horacek, 1973; Miller and Geselowitz, 1978; Mailloux and Gulrajani, 1982; Aoki et al., 1987).

The purpose of this paper is to show that a lead-field approach can greatly improve scalability in a high-performance computing (HPC) context without sacrificing accuracy. This is not obvious, because the method requires a large set of transfer coefficients (the lead field) to be stored between the two phases of the computation. The efficiency of the method depends on the accuracy with which the lead field must be computed and the degree to which it can be downsampled without affecting the accuracy of the ECG too much. Finally, to provide answers to these questions an accurate reference solution is needed.

Using a reference solution computed on a full torso model at 0.2 mm resolution this study shows that the lead field can indeed be downsampled enough to achieve an efficient and scalable computation, providing roughly two orders of magnitude speedup with negligible loss in accuracy.

The results of this study make it possible to build more realistic heart models with higher spatial resolution, without spending much more time to compute the ECG.

## 2. Methods

### 2.1. Model equations

The methods in this study are based on the bidomain model of cardiac electrophysiology (Miller and Geselowitz, 1978; Tung, 1978), on which most of the current modeling work in this area is based (Niederer et al., 2011a; Henriquez, 2014). The bidomain model is a continuum approximation of the heart muscle, which in reality consists of a network of interconnected muscle cells embedded in an extracellular matrix and other structures such as fibroblasts and capillaries. The bidomain model approximates this as two co-located spaces: the intracellular domain, consisting of the interior of the cells and the gap junctions that connect them, and the extracellular domain, consisting of everything else.

The two domains are characterized by conductivity tensors *G*_i_ and *G*_e_, respectively. Their values at each point in the model depend on the fiber direction and account for the partial volume occupation of the two domains. In addition the parameters *C*_m_ and β determine the capacitance of the cell membrane and the amount of membrane per unit volume, respectively. The state variables of the model are the potential fields ϕ_i_ in the intracellular and ϕ_e_ in the extracellular domain, and a set of variables $\vec{y}$ describing the state of the membrane model at each location. Using the auxiliary variable *V*_m_ = ϕ_i_ − ϕ_e_ and agreeing that all variables are functions of time and position we can express the bidomain model compactly as

(1) $$\beta^{-1}\nabla \cdot (G_{\text{i}}\nabla \phi_{\text{i}})=C_{\text{m}}\partial_{t}V_{\text{m}}+I_{\text{ion}}(V_{\text{m}},\vec{y})$$

(2) $$\beta^{-1}\nabla \cdot (G_{\text{e}}\nabla \phi_{\text{e}})=-C_{\text{m}}\partial_{t}V_{\text{m}}-I_{\text{ion}}(V_{\text{m}},\vec{y})$$

(3) $$\partial_{t}\vec{y}=F(V_{\text{m}},\vec{y})$$

where the term *C*_m_∂_*t*_*V*_m_ represents the capacitive transmembrane current, the function *I*_ion_ the density of ionic current flowing between the two domains, and *F* is a nonlinear vector-valued function describing how the membrane state evolves. The pair of functions *I*_ion_ and *F* constitutes the membrane model. Suitable boundary conditions are

(4) $$G_{\text{i}}\nabla \phi_{\text{i}}\cdot \partial \Omega_{\text{A}}=0$$

on the boundary Ω_A_ of the cardiac muscle and

(5) $$G_{\text{e}}\nabla \phi_{\text{e}}\cdot \partial \Omega_{\text{T}}=0$$

on the torso boundary Ω_T_ (Tung, 1978; Krassowska and Neu, 1994).

The electrical activity of the heart can then be simulated by integrating Equations (1), (2), and (3) under the boundary conditions (4) and (5) (Vigmond et al., 2002). This is known as a bidomain reaction-diffusion model. In this study a simplified version, a “monodomain” reaction-diffusion model, was used. This model can be derived by assuming that *G*_i_ and *G*_e_ are proportional (Leon and Horácek, 1991). Although this is a gross simplification the effect of this assumption is negligible for most purposes if the model parameters are well chosen (Potse et al., 2006; Nielsen et al., 2007; Bishop and Plank, 2011; Coudière et al., 2014). The monodomain model reads

(6) $$\{\begin{array}{l} C_{\text{m}}\partial_{t}V_{\text{m}}=\beta^{-1}\nabla \cdot \left(G_{\text{m}}\nabla V_{\text{m}}\right)-I_{\text{ion}}\left(V_{\text{m}},\vec{y}\right)\\ \partial_{t}\vec{y}=F\left(V_{\text{m}},\vec{y}\right) \end{array}$$

The “monodomain conductivity tensor” *G*_m_ was computed as the series conductivity of the two domains, *G*_m_ = *G*_i_*G*_e_/(*G*_i_ + *G*_e_). With this choice the resistance encountered by a current loop through the cell membrane is the same as in a bidomain model, so that also the conduction velocity of a propagating activation wavefront is almost the same.

An ECG potential *V*(*t*) at time *t* is the difference in ϕ_e_ between two locations on the body surface or, more generally, a linear combination

(7) $$V(t)=\sum_{i}c_{i}\phi_{\text{e}}^{i}$$

where *c*_*i*_ are the relative contributions of the two or more electrodes and $\phi_{\text{e}}^{i}$ are the potentials at the corresponding positions. The coefficients *c*_*i*_ must fulfill charge conservation, ∑*c*_*i*_ = 0.

To compute ϕ_e_ we must return to the bidomain model. Equations (1) and (2) can be combined and reorganized to yield

(8) $$\nabla \cdot ((G_{\text{i}}+G_{\text{e}})\nabla \phi_{\text{e}})=-\nabla \cdot (G_{\text{i}}\nabla V_{\text{m}}).$$

This equation can be solved for ϕ_e_ in the whole torso at once from a given distribution of *V*_m_. However, for the ECG we need to know ϕ_e_ at a few locations only. Therefore, it can be more efficient to use a Green's function of the operator ∇ · ((*G*_i_ + *G*_e_)∇.) for each of these locations. Since an ECG lead is a linear combination of ϕ_e_ at two or more points it can also be represented directly by a linear combination of Green's functions. In electrocardiology such linear combinations of Green's functions are named lead fields (McFee and Johnston, 1953; Geselowitz, 1989; Colli-Franzone et al., 2000). A lead field is computed once for each ECG lead. It is then used to evaluate the ECG at each time step of the reaction-diffusion model and, as long as the conductivity parameters are not changed, can be re-used for multiple simulations. In terms of a lead field $Z\left(\vec{x}\right)$ the ECG potential *V*(*t*) at time *t* is

(9) $$V(t)=\int \nabla Z(\vec{x})\cdot G_{\text{i}}\nabla V_{\text{m}}d\vec{x}$$

where the integration is over the myocardium. In contrast to the solution of the full system (8) this calculation is simple and *a priori* highly scalable. The lead field can be computed as the potential field resulting from a unit current applied at the electrode locations ${\vec{x}}_{i}$ (Geselowitz, 1989):

(10) $$\nabla \cdot ((G_{\text{i}}+G_{\text{e}})\nabla Z(\vec{x}))=\sum_{i}c_{i}\delta (\vec{x}-{\vec{x}}_{i})$$

where the coefficients *c*_*i*_ are as in Equation (7) and δ is Dirac's delta function. To avoid a scaling factor in (9) the total injected current must be unitary, ∑|*c*_*i*_| = 2.

### 2.2. Model geometry

In order to run tests on a relevant geometry a model of the heart and torso was used that had been created for a previous study (Kania et al., 2017). The methods to build this geometry, only tersely described before, were as follows. High-resolution cardiac and thoracic computed tomography (CT) images were obtained from a female patient in her thirties. Images were segmented automatically using the MUSIC software (IHU Liryc, Université de Bordeaux and Inria Sophia Antipolis, France), under supervision of an expert operator. The boundaries of the segmented volumes were expressed as triangulated surfaces and meshing errors were manually corrected using Blender (The Blender Foundation, Amsterdam, The Netherlands). The resulting surface mesh defined the volumes of the ventricular myocardium, left and right cavities with parts of the great vessels, lungs, and the whole body. To define hexahedral meshes for the computations the surfaces were overlaid with a 3D cartesian mesh whose elements were assigned types according to the surfaces in which they were contained. The bones were also segmented and meshed but not included in the simulations. The atrial myocardium was not segmented.

The heart mesh was processed to define subendocardial and subepicardial layers and fiber directions using the rule proposed by Beyar and Sideman (1984), as previously described (Potse et al., 2006). The torso mesh was similarly processed to define a layer of 1 cm thickness directly under the skin as skeletal muscle and to define a sheet direction in this layer. Since the true fiber directions of the skeletal muscle layer are too complex to account for the model muscle simply had a low conductivity in the radial direction and a high conductivity in all circumferential directions (Table 1).

**Table 1:** Tissues used in the simulations together with the volumes they occupy in the torso model, the conductivity parameters σ (in mS/cm), and β (cm^−1^); the subscript “i” stands for intracellular, “e” for extracellular, “L” for longitudinal, “T” for transverse (within a tissue sheet), and “C” for across-sheet.

**Material**	**Volume (mL)**	**σ_iL_**	**σ_iT_**	**σ_iC_**	**σ_eL_**	**σ_eT_**	**σ_eC_**	**β**
Myocardium	110	3.0	0.3	0.3	3.00	1.20	1.20	800
Body	16,482	0	0	0	2.00	2.00	2.00	0
Blood	236	0	0	0	6.00	6.00	6.00	0
Lung	4,352	0	0	0	0.50	0.50	0.50	0
Muscle	5,605	0	0	0	3.55	3.55	0.44	0

**Figure 1:**
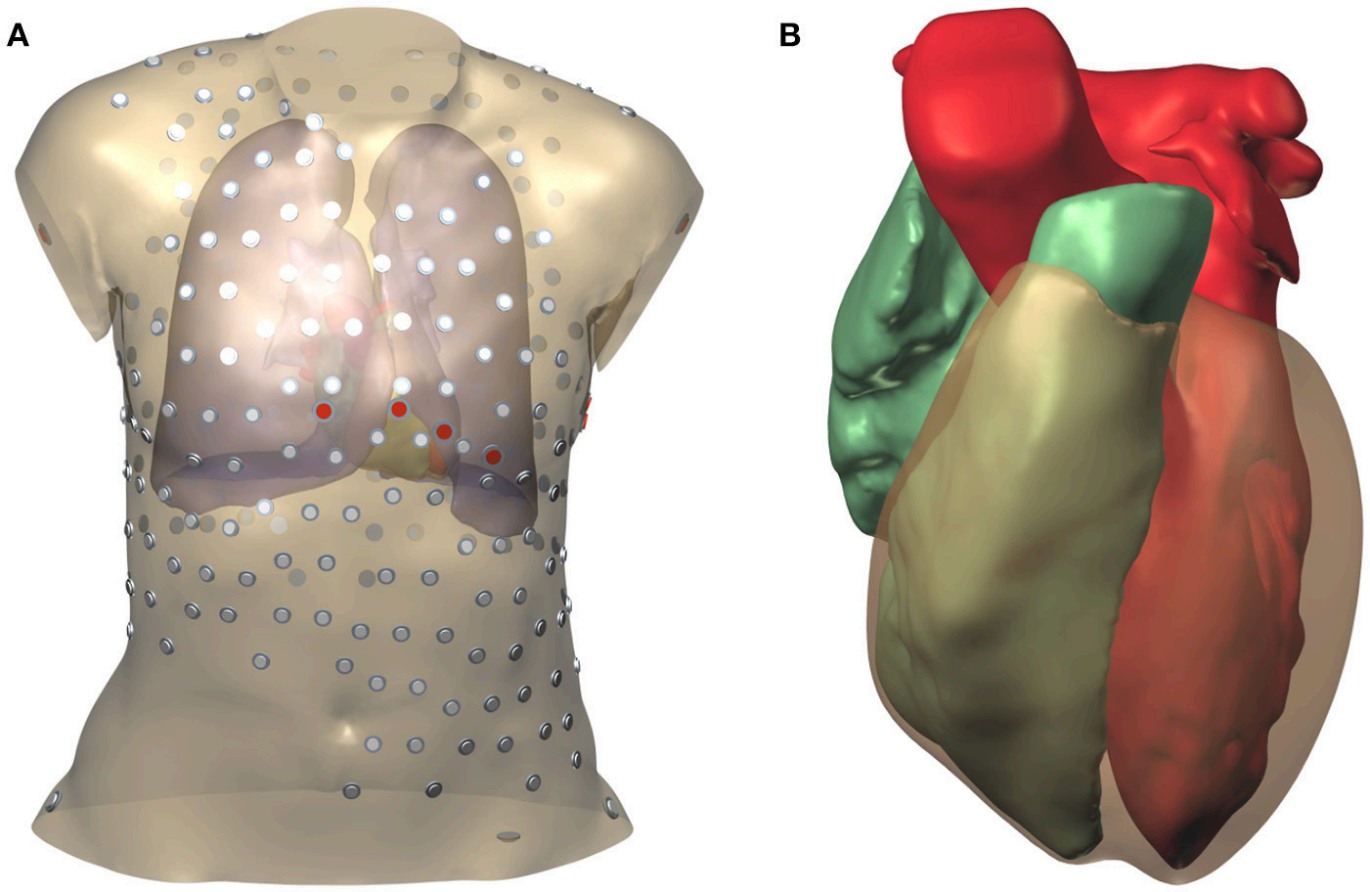
Model geometry and electrode positions. **(A)** Torso model. The smaller electrodes with a gray cap belong to the vest; those with a red cap are the standard ECG electrodes. **(B)** Surfaces representing the two cardiac cavities and the ventricular epicardium.

During the thoracic scan the patient was wearing a vest with 252 embedded electrodes (Tilt et al., 2013; Cochet et al., 2014). The locations of these electrodes were extracted from the CT data using software provided by the manufacturer of the vest. In addition the locations of the 9 standard ECG electrodes were determined by referring to the bone mesh, and two electrode locations on the hips were chosen. The surface mesh with electrode positions is illustrated in Figure 1.

### 2.3. Spatial discretization

Spatial discretization was done using a finite-difference method. Differential operators of the form ∇·(*G*∇.), where *G* is any of the conductivity tensor fields employed, were computed using an expression proposed by Saleheen and Ng (1997). This expression assumes that *G* is constant on elements and that potentials are defined on the nodes of the mesh. It produces a 19-point stencil that takes anisotropy and inhomogeneities into account. The simulation code read its geometry in terms of elements, and created a node mesh, assigning node types such that all corners of a myocardial element would have myocardial nodes. In order to treat myocardial boundaries correctly, the β value of each node was the average of those associated with the 8 elements around it, which was zero for non-myocardium (Potse et al., 2006).

### 2.4. Simulation of cardiac activity

To prepare input data for ECG simulation propagating activation was simulated using the monodomain reaction-diffusion model (6) using the membrane model of Ten Tusscher and Panfilov (2006) for the functions *F* and *I*_ion_. A uniform time step of 10 μs was used. At each time step the code

1. evaluated the diffusion current β^−1^∇·(*G*_m_∇*V*_m_Δ*V*_m_),
2. communicated the diffusion current across domain boundaries,
3. integrated the membrane status variables $\vec{y}$,
4. evaluated $I_{\text{ion}}\left(V_{\text{m}},\vec{y}\right)$, and
5. integrated *V*_m_.

After each 100 time steps results were written to file. Simulations were run on a heart mesh at 0.2 mm resolution. Tissue parameters determining *G*_m_ and β are listed in Table 1. Gating variables were integrated with the method of Rush and Larsen (1978) and all other variables with a forward Euler method.

Activation was started with a single stimulus at one location, at the beginning of the simulation. Seven simulations were run, each time with the stimulus at a different location. Simulations covered 500 ms to include the full depolarization and repolarization of the ventricles.

### 2.5. ECG simulation

The ECG was computed with several methods:

**FSF, the fine-mesh full solution** solved the full system (8) for given *V*_m_ on a heart-torso mesh with 0.2 mm resolution. This was an exceptionally large computation requiring 3.3·10^9^ mesh nodes and 12 TB memory. It was combined in a single run with the integration of the monodomain reaction-diffusion model (6). Solutions for ϕ_e_ were computed after each 100 time steps.
**FSC, the coarse-mesh full solution** solved an alternate form of Equation (8) on a heart-torso mesh with 1 mm resolution (Potse and Kuijpers, 2010). In this case the equation read
  (11) $$\nabla \cdot ((G_{\text{i}}+G_{\text{e}})\nabla \phi_{\text{e}})=-I_{\text{w}}$$
  where *I*_w_ is a projection of the term ∇·(*G*_i_∇*V*_m_) from a 0.2 mm resolution heart mesh onto a 1 mm resolution torso mesh. Each coarse-mesh node received contributions from a cube-shaped area including all fine-mesh nodes within the up to 8 coarse-mesh elements around it, with higher weights attributed to nearby nodes, as in a trilinear interpolation: Let Δ*x*, Δ*y*, Δ*z* be the number of fine-mesh edges between a coarse-mesh node and a fine-mesh node along the x, y, and z axis, respectively. Then the contribution of the fine-mesh node to the coarse-mesh node was
  $$w=\left\{ \begin{array}{l} 0,\text{ if }\Delta x\geq 5\vee \Delta y\geq 5\vee \Delta z\geq 5\\ (5-\Delta x)(5-\Delta y)(5-\Delta z)/5^{6},\text{ otherwise.} \end{array}\right.$$
  The coarse mesh was constructed such that a myocardial fine-mesh node was always surrounded by 8 coarse-mesh nodes. Therefore, *w* added up to unity for each fine-mesh node and charge conservation was ensured.
  For the FSC method the monodomain reaction-diffusion model (6) was integrated in a separate run which saved *I*_w_ to file. This method has been used routinely in several studies (Nguyên et al., 2015; Meijborg et al., 2016; Duchateau et al., 2017; Kania et al., 2017). The torso mesh in this case consisted of 2.7·10^7^ nodes.
**LF, the lead-field method** evaluated the integral expression (9) in its discretized form. This took place during the reaction-diffusion simulation and on the same mesh, i.e., at 0.2 mm resolution, after each 100 time steps. Each component of ∇*V*_m_ was evaluated on model elements as an average of the differentials along 4 edges of the element. The conductivity tensor *G*_i_ was also evaluated on each element. For testing purposes the lead vector field ∇*Z* was evaluated at different resolutions. For this purpose the field was first downsampled by an external program, using a simple averaging of *n* × *n* × *n* elements, where *n* could be 2, 5, 10, or 25.
**LFS, the lead-field method with selective downsampling** was identical to the lead-field method except that the downsampling program took the tissue types of the elements into account. If any of the fine-mesh elements inside a coarse-mesh element *E* had a myocardial type, only fine-mesh elements with myocardial type were used in the average for *E*. The idea behind this was that ∇*Z* undergoes abrupt changes at the myocardial boundaries, and that it is more accurate to mix in a contribution from another myocardial area than, for example, one from the lung.

The notations LF(*C, S*) and LFS(*C, S*) will be used for the LF and LFS methods, respectively, with lead fields computed at a resolution of *C* millimeters and downsampled to a resolution of *S* millimeters.

### 2.6. Computation of lead fields

To prepare the lead fields *Z* for the ECG computation the system (10) was solved for each lead. This was done once with a torso model at 1 mm resolution and once with a torso model at 0.2 mm resolution. Like the FSF, the latter calculation was exceptionally large and was only intended to provide reference values, to test the hypothesis that 1 mm resolution suffices for such calculations.

In either case 266 lead fields were computed: the 12 standard ECG leads, and one lead for each of the 252 vest electrodes and 2 hip electrodes referenced against Wilson's central terminal (the average of the two arm electrodes and the left leg electrode).

The computed lead fields *Z* were stored in files. A dedicated program computed ∇*Z* and downsampled it using the two methods described in section 2.5, i.e., with and without consideration of the tissue types of the elements. The field computed at 0.2 mm resolution was downsampled by the factors 2, 5, 10, and 25 to obtain resolutions of 0.4, 1, 2, and 5 mm. The field computed at 1 mm resolution was downsampled by the factors 2 and 5 to obtain resolutions of 2 and 5 mm.

### 2.7. Testing protocol

ECGs were simulated using each of the 4 methods described in section 2.5 and, for the methods based on lead fields, at each of the resolutions mentioned in section 2.6.

The ECG potentials *V* were compared to a reference ECG *V*^ref^ in terms of three measures: maximum, root-mean-square (RMS), and relative difference (RelDif) (van Oosterom, 2001; Tysler et al., 2007), defined as

(12) $$\text{RelDif}=\sqrt{\frac{\sum_{t}\sum_{n}{\left(V_{tn}-V_{tn}^{\text{ref}}\right)}^{2}}{\sum_{t}\sum_{n}{\left(V_{tn}^{\text{ref}}\right)}^{2}}}$$

where the index *t* ranges over all 500 samples and the index *n* ranges over all 266 leads. For the 252 vest leads the dependence of the error values on the position of the positive electrode was investigated.

The effect of the ECG computation on the run time of a reaction-diffusion model was investigated and the scalability of the 4 methods was investigated by running tests on 16, 32, …, 512 nodes of a Bull cluster. Each of these nodes was equipped with two 14-core Intel Xeon E5-2690 processors with 2.6 GHz clock frequency and 64 GB memory. Accuracy results are reported as averages over the 7 activation sequences. Performance tests were carried out 5 times to report average values and standard deviations of run time.

### 2.8. Numerical methods

Simulations were performed using the Propag-5 software (Krause et al., 2012), to which new code was added to compute a lead field-based ECG on the fly during a simulation of the heart, and to facilitate the computation of the lead fields themselves. Like its predecessor Propag-4 (Potse et al., 2006), the software uses a structured mesh, but stores information only for elements and nodes that are relevant for the computation: only myocardium for a monodomain model, and only conducting material for a bidomain model. As discussed by Krause et al. (2012) Propag-5 uses a hybrid MPI/OpenMP parallellization scheme. Using a naive temporary partitioning of the domain the code reads the geometry in terms of elements and creates a node mesh using rules that ensure consistency with the scheme discussed in section 2.3. It then uses the ParMetis library to partition this mesh in parallel and creates a definitive domain partitioning for the computations. This fully parallel workflow allowed it to load and partition a mesh with over 3 billion nodes.

Because in some of the computations the model size exceeded the maximum value of a signed 32-bit integer, Propag was compiled with a 64-bit integer type for global indices. The PetSC (Balay et al., 2017) and Parmetis libraries which Propag uses were compiled entirely with 64-bit integers because they do not have a distinct type for global indices.

The linear systems (8), (10), and (11) were solved with a biCGStab solver (van der Vorst, 1992) with a BoomerAMG preconditioner from the Hypre package (Henson and Meier Yang, 2002; Falgout et al., 2017). The solver terminated when the norm of the error term was 10^−8^ times smaller than the norm of the right-hand side. Multigrid preconditioners such as BoomerAMG are very powerful and well-suited for large bidomain problems (Sundnes et al., 2002; Weber dos Santos et al., 2004; Austin et al., 2006) so that the solver typically needs only a handful of iterations, in contrast to the problematic convergence observed on large models with an incomplete-LU preconditioner (Potse et al., 2006).

## 3. Results

**Figure 2:**
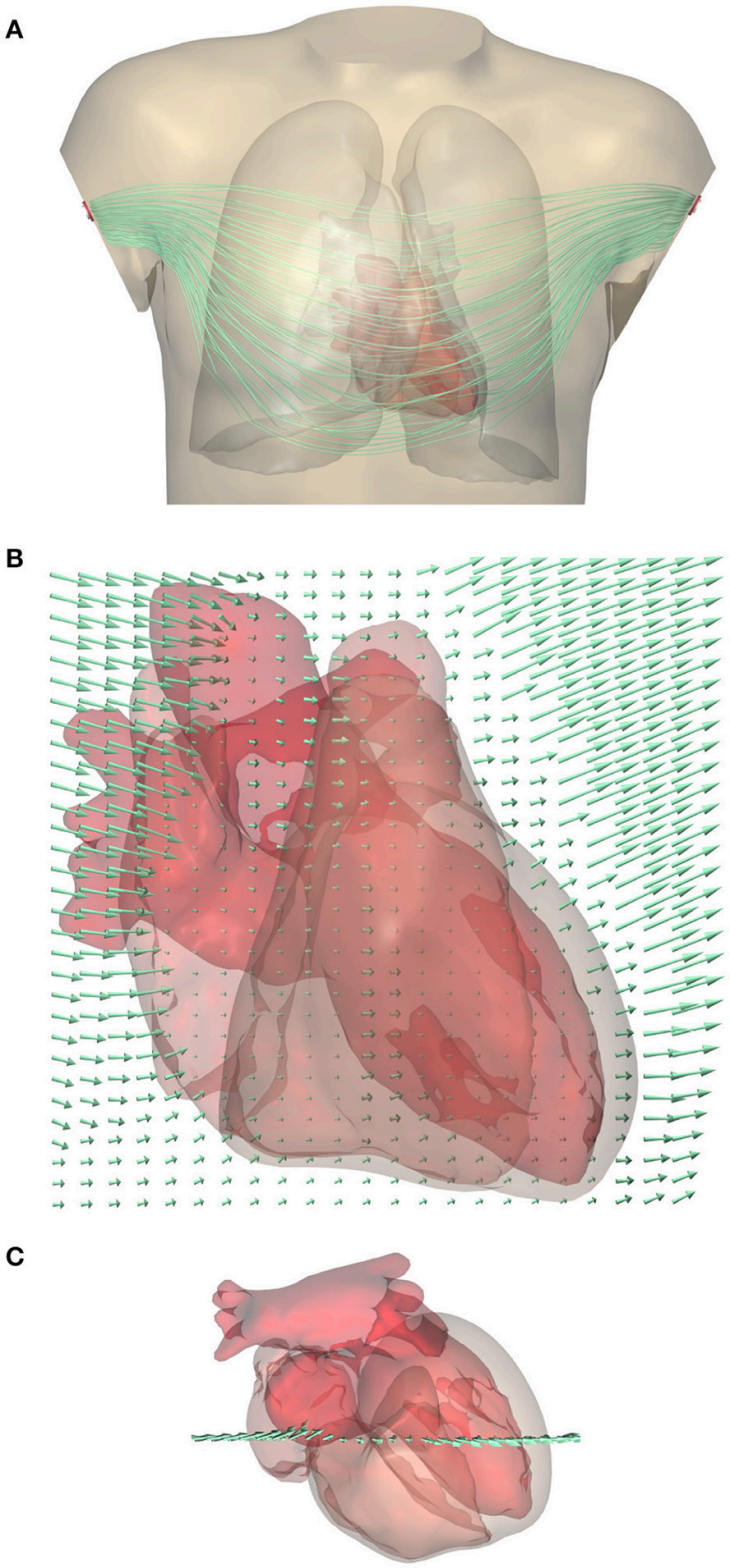
**(A)** Computed lead field for standard lead I (between the two arm electrodes) shown using streamlines that represent the direction of ∇*Z*. Note that they do not represent the current (*G*_i_+*G*_e_)∇*Z*. For a clear visualization the lines were seeded at a selection of points in and near the heart; variations in field strength perpendicular to the lines cannot be read from this figure. A rotating display is provided in a Supplementary File. **(B)** Close-up of the lead field on a grid in a frontal plane crossing the heart. The arrow length shows the strength of the field, |∇*Z*|, at the tail of the arrow. Field strength is small in the highly conductive blood inside the ventricles, and very large in the low-conductivity lungs, while it has an intermediate value in the cardiac muscle and in the abdomen. **(C)** Superior view of the heart showing the location of the grid.

An example of a computed lead field is shown in Figure 2. This field was computed and stored at 1-mm resolution. The figure shows how the field suddenly changes direction and magnitude at lung boundaries. There is a slight left-right asymmetry because the highly conductive cardiac cavities concentrate the field on the left side of the thorax.

**Figure 3:**
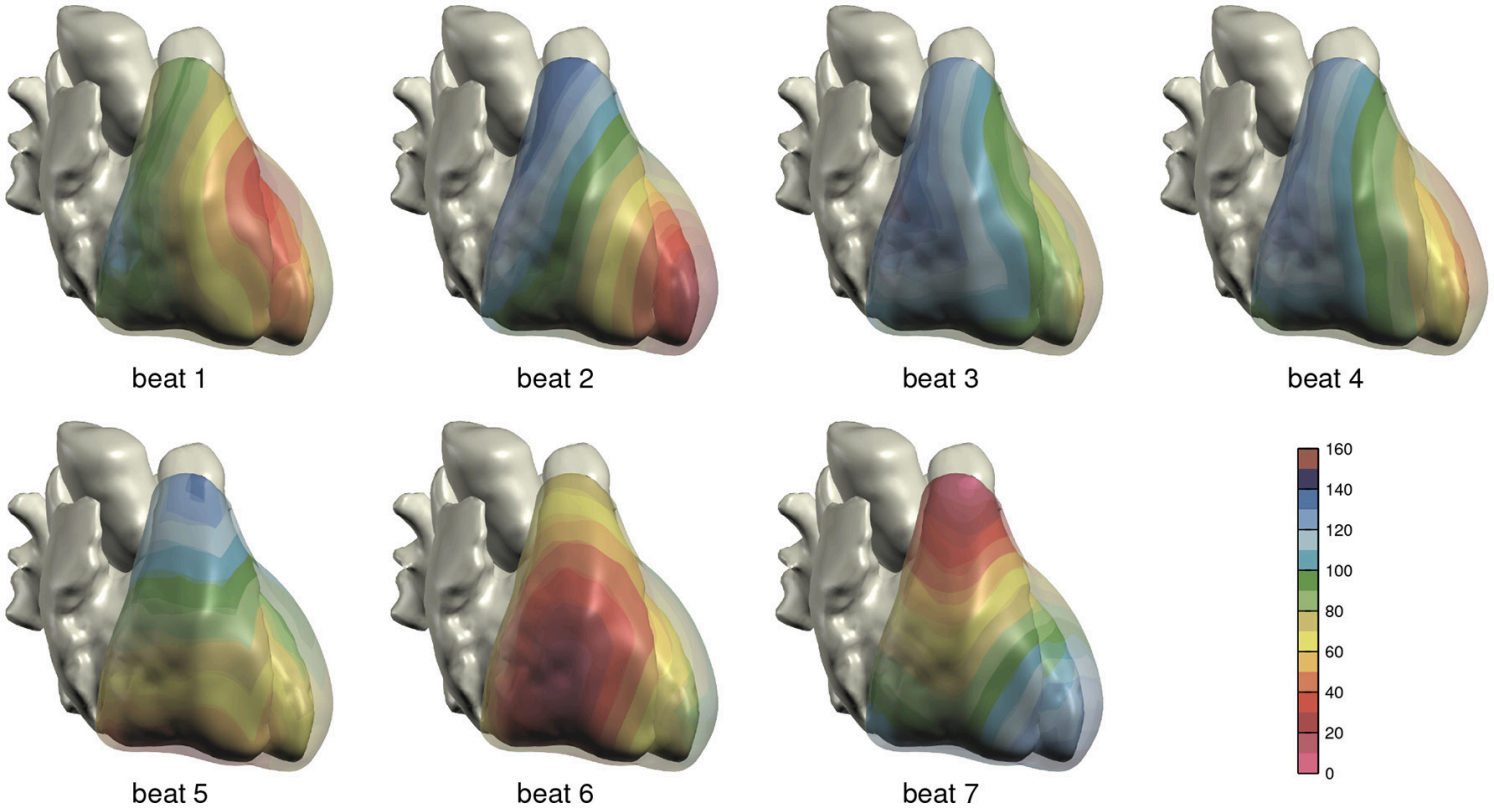
Depolarization order in the 7 monodomain reaction-diffusion simulations from which ECGs were computed; anterior view. The scale is in milliseconds.

The computed depolarization sequences of the 7 simulated heart beats that were used for ECG computation are shown in Figure 3.

**Figure 4:**
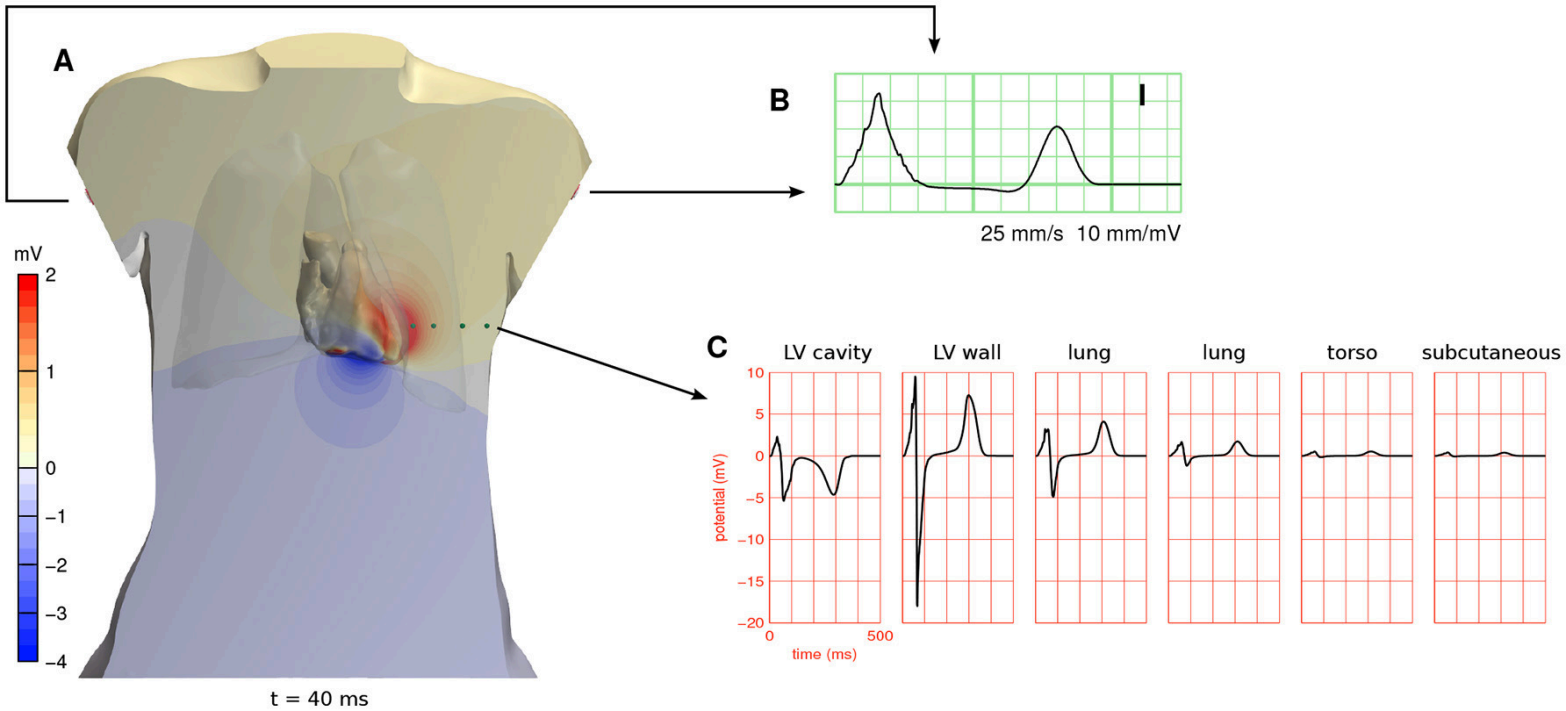
**(A)** Full-torso solution for the potential field ϕ_e_ 40 ms after the start of the simulation in beat 5 (see Figure 3), shown in a cross section of the torso and on the surface of the cardiac cavities. A movie showing the potential field throughout the heart beat is provided in a Supplementary File. **(B)** ECG lead I, which is measured between the two arm electrodes, from the same simulation. **(C)** ϕ_e_ at 6 positions in the cross-sectional plane of **(A)**. The first position was inside the left ventricular cavity and the second in the left-ventricular free wall. The other four are marked with green spheres. These potentials are referenced against Wilson's central terminal (the average potential of the three limb electrodes).

Potentials computed with a full-torso solution from beat 5 are shown in Figure 4. They are about 10 times larger in the myocardium than near the body surface.

### 3.1. Lead-field ECG compared to full solution

To establish that the lead-field and full solution methods produce the same results, simulated ECGs were compared between the LF(1, 1) and FSC methods. Averaged over the 7 simulations, RelDif was 0.0016, RMS error 0.3 μV, and maximum error 4.6 μV, while ECG amplitudes were in the order of 1 mV.

Analogously, a single ECG was compared between the LF(0.2, 0.2) and FSF methods. In this case the differences were slightly smaller: RelDif was 0.0014, RMS error 0.2 μV, and maximum error 2.6 μV.

### 3.2. Effect of resolution

To determine the effect of lead-field resolution on ECG accuracy, 7 different activation sequences were simulated with a monodomain reaction-diffusion model and ECGs were simulated on the fly using a lead field. This was done for the lead fields computed at 0.2 and at 1.0 mm and all downsamplings thereof, both with the LF and with the LFS method. The resulting ECGs were compared to a reference ECG.

**Figure 5:**
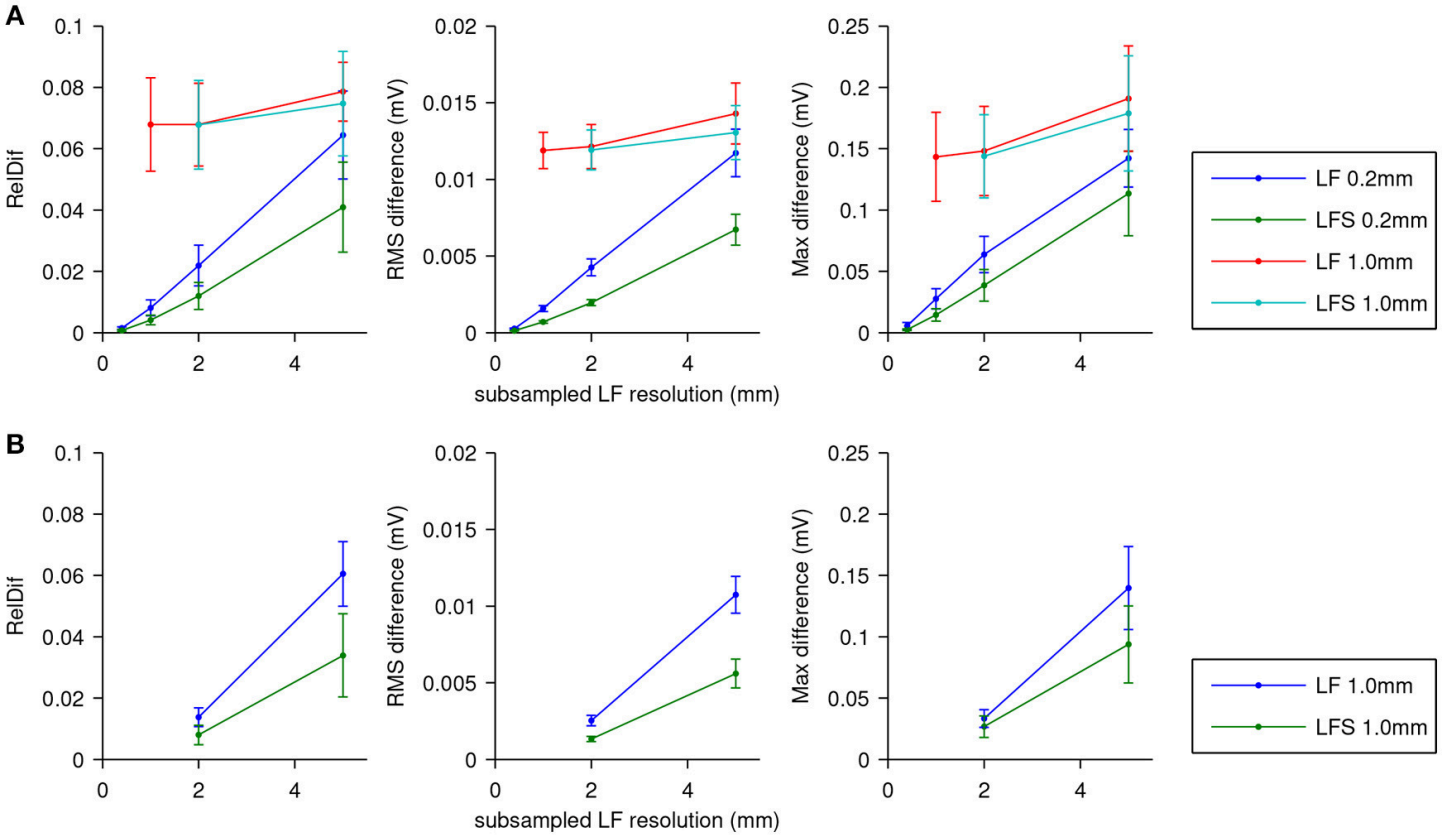
ECG computation error expressed as RelDif, RMS difference, and maximum difference. In each panel dots indicate average values and the whiskers indicate standard deviations of each statistic over the 7 activation sequences. **(A)** Error for the LF and LFS methods with fields downsampled from those computed at 0.2 and 1.0 mm resolution, using the field at the full 0.2 mm resolution as the reference. **(B)** Error for the LF and LFS methods with only fields downsampled from the one computed at 1.0 mm resolution, using the field at 1.0 mm resolution as the reference.

The results are shown in Figure 5. In Figure 5A errors are shown using the ECG computed with LF(0.2, 0.2) as the reference. For the fields subsampled from those computed at 0.2 mm resolution, differences are seen to increase roughly linearly with the stepsize of the lead field. The LFS method resulted in smaller differences. Results obtained with the field computed at 1.0 mm resolution and downsamplings differed from the reference solution with little dependence on the sampling level. Figure 5B shows that this dependency is recovered when ECGs computed with LF(1, 1) are used as the reference.

The relatively large influence of the spatial stepsize in the lead-field computation suggests that differences in model geometry dominate the error. Indeed, the difference between full solutions at 0.2 and 1.0 mm, computed only for one simulation, had a RelDif of 0.10, RMS error 12 μV, and maximum error 0.15 mV, which are very similar to the differences between LF(1, 1) and LF(0.2, 1) in Figure 5A.

**Figure 6:**
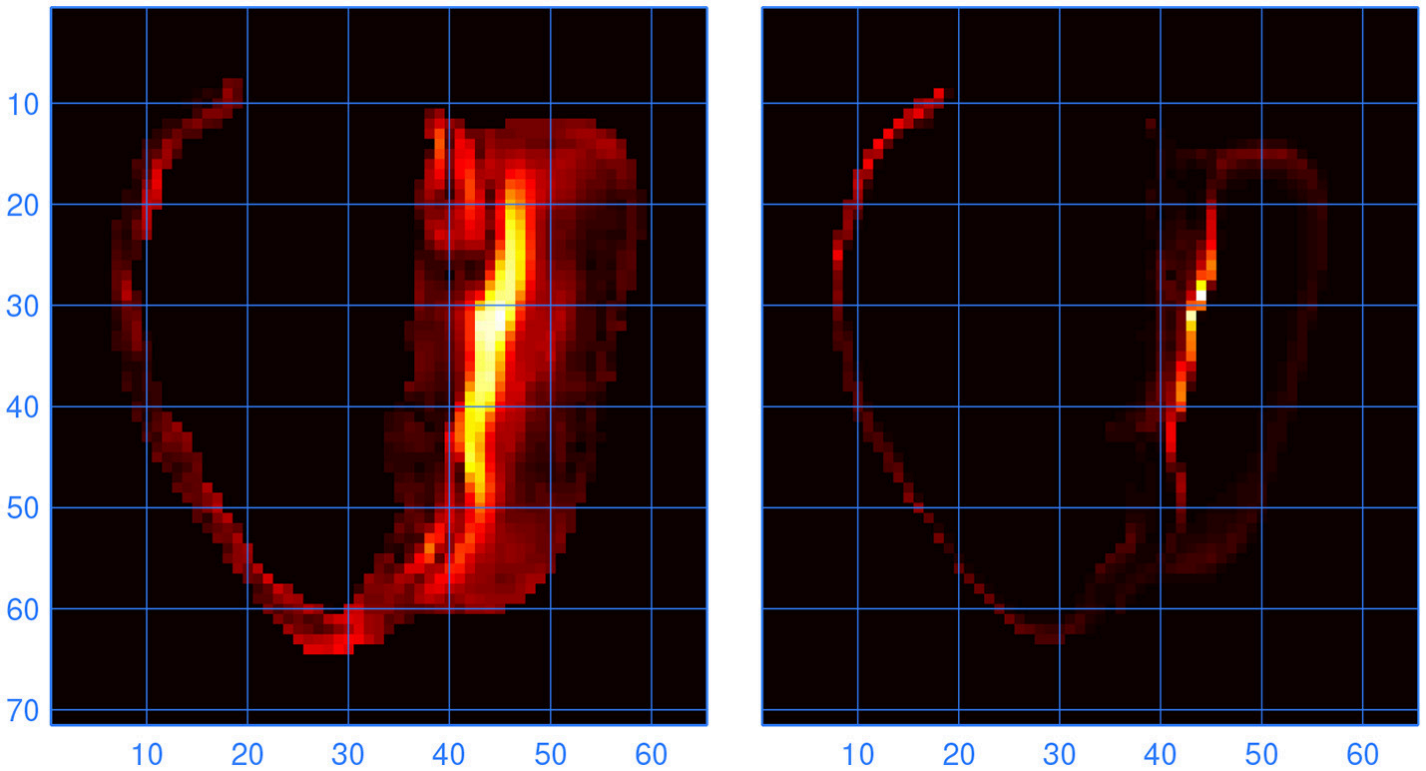
**(Left)** Norm of the difference between the lead vector fields computed with LFS(0.2, 1) and LF(1, 1). Brighter colors indicate higher values; the units are arbitrary. The cross section is through the inferior septal junction, parallel to the standard long-axis plane. The cavity on the left side of the image is the bulbus region of the right ventricle. **(Right)** Variability in fiber direction, also in arbitrary units, in the same cross section. The scales are in millimeters.

To find out at which locations in the model the lead fields computed with LFS(0.2, 1) and LF(1, 1) differed, the L2 norm of the difference between the two vector fields was computed for all elements. Large differences were found to occur at locations where the fiber direction was highly variable. One such location, at the inferior septal junction, is illustrated in Figure 6. It is compared with a measure of variability in fiber direction in the underlying anatomy files, computed as

$$1-\frac{1}{N}\overset{N}{\underset{i=1}{\sum }}|\vec{P}\cdot {\vec{p}}_{i}|$$

where $\vec{P}$ is the fiber direction in the coarse-mesh element and ${\vec{p}}_{i}$ are the fiber directions in the corresponding fine-mesh elements. The absolute value, denoted as |.|, was taken because the orientation of the direction vector is irrelevant.

**Figure 7:**
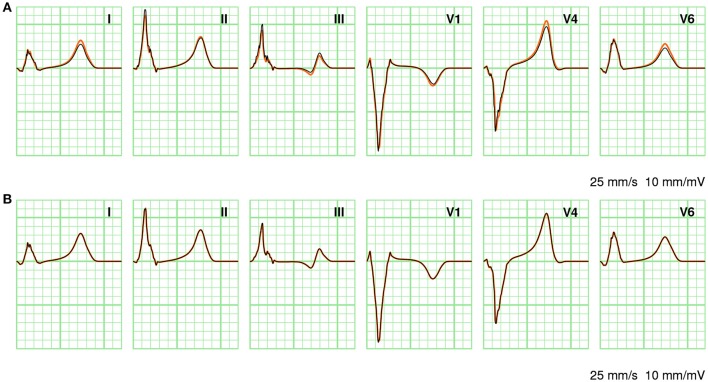
Comparison of ECGs between different computation methods. **(A)** Full solutions at 0.2 mm (orange) and 1.0 mm (black). **(B)** Full solution at 0.2 mm (orange) and LFS(0.2, 2). The standard limb leads I, II, and III as well as three standard precordial leads V1, V4, and V6 are shown. Vertical grid lines are 40 ms apart and horizontal grid lines are 0.1 mV apart.

In Figure 7 a few ECG leads are compared between different computation methods. In Figure 7A full solutions at 0.2 and 1.0 mm are compared. At the coarser resolution the ECG appears more fractionated; this is particularly visible in lead III. As discussed above, the RelDif between these ECGs was 0.10. In Figure 7B the same full solution at 0.2 mm is compared with an ECG computed with LFS(0.2, 2). Despite the 10-fold downsampling of the lead field the traces are visually identical; the RelDif was 0.02. Thus, an ECG computed with a lead field downsampled to 2 mm resolution is more faithful than a full solution at 1 mm resolution, when compared to a solution at 0.2 mm.

### 3.3. Performance

Table 2 shows how ECG computation with lead fields at different resolutions affects the run time of a typical simulation. The data in each row were obtained from 5 simulations of 500 ms activity with a reaction-diffusion model at 0.2 mm resolution, run on 32 compute nodes (896 cores). The table separates initialization time, ECG computation time, and simulation time (including ECG computation but excluding initialization). For lead fields at 0.2 and 0.4 mm resolution the initialization time is of the same order of magnitude as the simulation time, due to the time it takes to read the lead fields from file (141 and 53 GB in these cases). The time for ECG computation itself ranges between 4 and 5 % of the simulation time, slightly reducing with the lead-field resolution. At 1 mm resolution the memory accesses related to ∇*Z* (for 266 leads) are similar to those for *G*_i_∇*V*_m_ so a further reduction would not be expected. At 0.2 mm resolution the ECG computation is faster than at 0.4 mm, likely because in this case the lead field has the same resolution as the reaction-diffusion model and the code then avoids an index conversion.

**Table 2:** Time required for LF-based ECG computation during a reaction-diffusion simulation of 500 ms.

**res**	**sim**	**ECG**	**init**
0.2	172.6 ± 0.7	8.3 ± 0.0	216.2 ± 3.6
0.4	179.2 ± 0.4	9.8 ± 0.1	103.3 ± 1.9
1.0	173.5 ± 1.8	8.1 ± 0.1	28.2 ± 1.5
2.0	171.8 ± 1.4	6.9 ± 0.3	25.8 ± 1.0
5.0	171.3 ± 2.2	6.2 ± 0.2	14.5 ± 0.7

*res, lead-field resolution in mm; sim, total simulation time; init, initialization time. Time is given as average ± standard deviation over 5 simulations, in seconds*.

**Figure 8:**
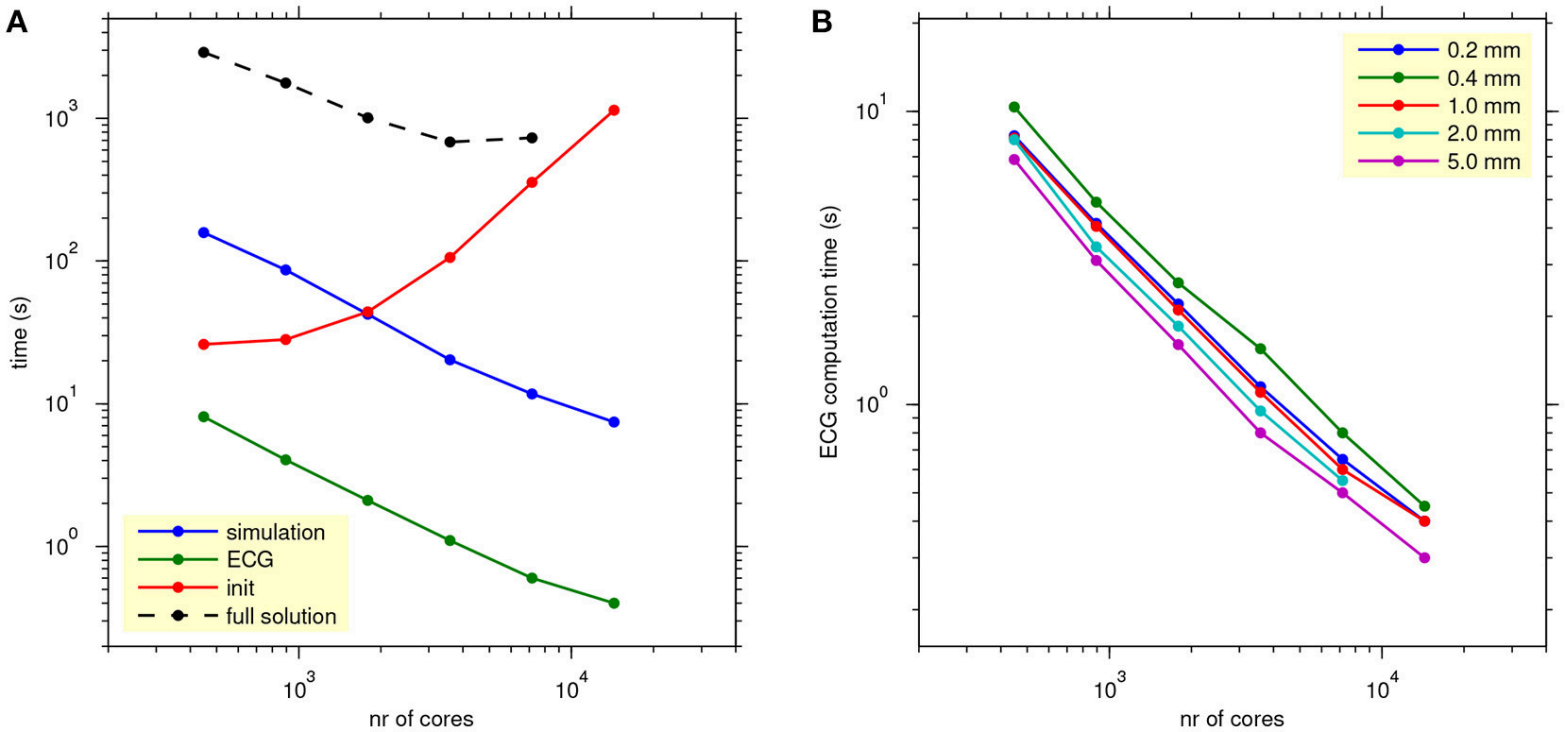
**(A)** Scaling of propagation, lead-field ECG, and full solution. The blue, green, and red traces show average simulation time, ECG computation time, and initialization time for reaction-diffusion simulations run on 16–512 nodes (448–14,336 cores) with 4 threads per process, with ECG computation based on a lead field at 1.0 mm resolution. The black trace shows the time for a full bidomain solution. Each data point represents an average over 5 simulations. **(B)** As **(A)**, but showing only ECG computation time, for all lead-field resolutions.

Figure 8A, shows how the computation times scale with the number of cores used for a single lead-field resolution of 1.0 mm. The reaction-diffusion simulation and the ECG computation scale well. Initialization time increases with the number of cores, due to increasing communication for mesh distribution and data input. Tests with higher and lower lead-field resolutions, not presented in the figure, showed that the initialization time was highly variable and had no clear relation with the resolution (and thus the storage size) of the field. Rather, the number of collective read operations seemed to be determining.

The black trace in Figure 8A shows the scaling of a full solution (FSC method). It is over 2 orders of magnitude slower than the lead-field ECG and stops scaling at 7,168 cores.

Figure 8B shows how the ECG computation time scales with the number of nodes for all tested values of lead-field resolution. Lead-field resolution is seen not to affect the scaling with the number of cores. Generally the time decreases slightly with decreasing resolution but, as in Table 2, the computation at 0.2 mm was faster than the one at 0.4 mm.

## 4. Discussion

This study shows that a lead-field approach is an attractive solution for ECG simulation on (large) parallel computers whenever the number of ECG leads is smaller than the number of samples. It is about 100 times faster than a full solution, scalable to more than 10^4^ cores, and does not cause a significant loss in accuracy. Lead fields can be stored at a resolution as low as 2 mm, meaning that they do not use excessive disk space even for a few hundred leads.

### 4.1. Previous work on lead fields

The concept of lead fields was initially proposed by McFee and Johnston (1953) as a method to understand how ECG leads “view” the heart. Their purpose was in the first place to design leads that would be better in the sense that their fields would be more uniform inside the heart muscle (McFee and Johnston, 1954). Later the idea has been adopted for the purpose of accurate numerical simulation of the ECG (Geselowitz, 1989) and even local electrograms inside the heart (Colli-Franzone et al., 2000; Western et al., 2015).

The idea to use lead-field methods for ECG simulation has been widely adopted. While the very earliest studies did not use them, for example because they computed only a small number of potential distributions (Gelernter and Swihart, 1964) or because a full solution required less memory (Barr et al., 1966; Barnard et al., 1967), numerous studies are based on some form of lead fields or transfer coefficients between *V*_m_ in the heart and ϕ_e_ on the body surface (Horacek, 1973; Miller and Geselowitz, 1978; Mailloux and Gulrajani, 1982; Aoki et al., 1987; Lorange and Gulrajani, 1993; Trudel et al., 2004).

Mailloux and Gulrajani (1982) and further work from the same group (Lorange and Gulrajani, 1993; Trudel et al., 2004) used transfer coefficients that are mathematically identical to lead fields. Their transfer coefficients were computed with a boundary element model (BEM) which accounted for heterogeneity of the torso, but not for anisotropy. They found that they needed <100 regions to define these coefficients, likely because their model was isotropic. In the anisotropic model used here the lead field changed considerably through the wall, requiring a much higher though not prohibitive resolution. Jacquemet (2015, 2017) evaluated the performance of the same (BEM-based) method on a reaction-diffusion model of the human atria and found that 1,000 regions sufficed for a 1% accuracy.

Boulakia et al. (2010) reported that an ECG simulation based on a transfer matrix was 60 times faster than solving a coupled heart-torso problem. They were using a finite-element model with about 1 million tetrahedra whose sizes gradually increased from the heart to the torso surface, and a serial code. Despite the obvious differences in methods the speedup was very similar to what was found in the current study.

Electrocardiographic inverse modeling studies that used volumetric transmembrane potentials or current dipoles as their source models have also used transfer coefficients that are similar to lead fields (Liu et al., 2006; Wang L. et al., 2013).

### 4.2. Other methods to compute the ECG

Many other studies have used full torso solutions to obtain the ECG from a reaction-diffusion model using finite-difference (Potse et al., 2009; Hoogendijk et al., 2010; Meijborg et al., 2016; Chamorro-Servent et al., 2017) or finite-element models (Lines et al., 2003; MacLachlan et al., 2005; Boulakia et al., 2010; Keller et al., 2010; Zemzemi et al., 2015; Janssen et al., 2017). In some cases this was done because intracardiac electrograms in a torso-coupled heart were also simulated (Hoogendijk et al., 2010; Meijborg et al., 2016). The ECG is then a free by-product.

An interesting alternative is a mixed approach in which anisotropic regions such as the heart and skeletal muscle are handled with finite elements and isotropic regions with boundary elements (Pullan and Bradley, 1996), resulting in fewer degrees of freedom than a complete volume discretization.

There is a considerable body of literature dedicated to the problem of solving body-surface potentials from epicardial (extracellular) potentials (Barr et al., 1977; Pilkington et al., 1987; Stenroos and Haueisen, 2008), which has found an application in cardiac inverse modeling (Greensite and Huiskamp, 1998; Ramanathan et al., 2004; Shou et al., 2008). A formulation in terms of transmembrane potentials on the (endocardial and epicardial) surface of the cardiac muscle is possible if equal anisotropy of the intracellular and extracellular domain is assumed (Geselowitz, 1989; van Oosterom and Jacquemet, 2005) and is also used to solve cardiac inverse problems (Oosterhoff et al., 2016).

### 4.3. Strengths and limitations

ECG simulation based on lead fields is very fast and as scalable as a monodomain reaction-diffusion model. This makes it suitable for inclusion in the same model run on a large-scale parallel computer or a GPGPU, in contrast to full solutions, which would limit the scalability of the entire computation. This advantage is present whenever the number of ECG samples to be simulated exceeds the number of leads.

Lead-field methods can also be used to compute local electrograms in the heart but this may require a higher spatial resolution at least near the electrode (Colli-Franzone et al., 2000).

For detailed spatial mapping of potentials, either in the heart or on the torso surface, lead-field methods are less advantageous, as the number of locations might exceed the number of samples and may even be so large that the storage of the lead fields becomes a performance bottleneck. In such cases full solutions remain the method of choice and a relatively long solution time will have to be accepted. Although new developments in scalable preconditioners may improve the situation somewhat (Munteanu et al., 2009; Ottino and Scacchi, 2015), it is unlikely that full solvers will ever scale as well as an ECG computation based on lead fields.

It would also be challenging to use a lead-field approach in an electromechanical, deforming heart model. A lead field that would be deformed with the mesh might be a reasonable approximation but this has not been tested here.

The results of this study also suggest further improvements, in the first place the use of non-uniform mesh density for lead-field computation. Comparison of ECGs computed at 0.2 and 1.0 mm resolution showed that the latter had artefactual notches of about 0.05 mV amplitude in the QRS complex, due to misrepresentation of fiber orientation at locations where this orientation changed rapidly. This applied to both full solutions and lead-field ECGs. To avoid such artifacts one could try to ensure a smooth fiber orientation throughout the model (Bayer et al., 2012), but this can be challenging at the interventricular junctions, or whenever measured fiber orientations rather than rule-based orientations are used. The only alternative seems to be computation of the lead field with a mesh at the same resolution as the reaction-diffusion model inside the heart, and for improved efficiency a lower resolution elsewhere in the torso (Pullan and Bradley, 1996; Boulakia et al., 2010). While the computations could still be hard on a mesh with a wide variation in element size, the memory requirements would be much lower than the 12 TB reported here for the reference torso model.

Another possible improvement that would be relevant for very accurate computations with high-resolution lead fields is to develop suitable compression methods for lead-field data. Very likely the regularity of the field could be exploited by using fixed-point numbers in combination with spatial differentiation and a variable-length encoding.

In Figure 8A, a particularly unfavorable scaling of the initialization phase was shown for the propagation model with lead-field ECG. This was probably due to an issue with the collective reading operation in the MPI library that was used, but also to the fact that for this feasibility study little care had been taken to organize this efficiently—after all the specifications for this code depended on the outcome of the study. With these results in hand it should be possible to avoid this problem by using a more efficient storage format and organizing the read operation in a different way. The figure also shows that the FSC method takes an order of magnitude more time than the reaction-diffusion model. This difference is partly due to the small solver tolerance that was chosen for this study.

### 4.4. Applications

The use of lead-field methods simplifies the workflow for large-scale cardiac simulations, as it allows the ECG to be computed on the fly with very little overhead during a reaction-diffusion simulation on a mesh of the heart alone. Moreover, its high scalability allows the resolution of the models to be increased without causing a disproportional increase in the time needed for ECG computation.

The results of this study are not only relevant for work on large-scale computers but also for simulations on general-purpose graphics processing units (GPGPU). Reaction-diffusion simulations on GPGPUs have been reported by several groups (e.g., Bartocci et al., 2011; Neic et al., 2012; Mena et al., 2015; Kudryashova et al., 2017), recently even for a whole human heart model run on a desktop computer (Vandersickel et al., 2016). The strength of a GPGPU is that it provides thousands of parallel processors for the price of a single CPU. However, communication between these processors is a distinct weakness. With a method based on lead fields it is nevertheless possible to add rapid ECG computation to a model running on a GPGPU. Pezzuto et al. (2017) have recently reported such a method, though in combination with an eikonal model rather than a reaction-diffusion model.

In the context of ECG inverse models and model personalization a variety of methods has been reported ranging from infinite-medium potentials (Giffard-Roisin et al., 2017; Neic et al., 2017) to full-torso bidomain solutions (Wang D. et al., 2013). A lead-field approach could offer a solution that combines the speed of the former (if the computation of the lead field itself is excluded) with the accuracy of the latter. Only methods based on equivalent double layers (Geselowitz, 1992; van Oosterom and Jacquemet, 2005) offer more efficiency as they need to evaluate only the surface of the heart, but the price for this efficiency is that these methods neglect anisotropy. A lead-field approach combined with an eikonal-diffusion model for cardiac propagation (Konukoglu et al., 2011; Jacquemet, 2012; Neic et al., 2017) could soon be a practical solution for ECG inverse problems with an accuracy very close to the state of the art in forward modeling of the ECG.

## 5. Conclusion

Lead fields are a practical alternative for full-torso solutions when the number of ECG leads that need to be simulated is smaller than the total number of samples that will be calculated. The method is fast and highly scalable. Lead fields can be stored at a resolution as low as 2 mm without unacceptable loss of accuracy.

## Author contributions

The author confirms being the sole contributor of this work and approved it for publication.

### Conflict of interest statement

The author declares that the research was conducted in the absence of any commercial or financial relationships that could be construed as a potential conflict of interest.
